# Baron Liebig

**Published:** 1865-02

**Authors:** 


Baron Liebig.—Herr Von Liebig, the well-known chemist, has resigned his Chair in the University of Munich. He has received a brilliant offer from the corporation of London to superintend the disinfection, and application to agriculture, of the liquid and solid dejections of that immense city.

